# Time of Day Preferences and Daily Temporal Consistency for Predicting the Sustained Use of a Commercial Meditation App: Longitudinal Observational Study

**DOI:** 10.2196/42482

**Published:** 2023-04-10

**Authors:** Vincent Berardi, Rylan Fowers, Gavriella Rubin, Chad Stecher

**Affiliations:** 1 Department of Psychology Chapman University Orange, CA United States; 2 College of Health Solutions Arizona State University Phoenix, AZ United States; 3 Division of Behavioral & Organizational Sciences Claremont Graduate University Claremont, CA United States

**Keywords:** behavioral habits, habit formation, mindfulness meditation, mobile health, health app, app usage, meditation app, temporal analysis, circadian rhythm, healthy life style, physical activity, mental well being, habit, mindfulness, meditation, wellbeing, mental health, longitudinal, observational, advice, morning

## Abstract

**Background:**

The intensive data typically collected by mobile health (mHealth) apps allows factors associated with persistent use to be investigated, which is an important objective given users’ well-known struggles with sustaining healthy behavior.

**Objective:**

Data from a commercial meditation app (n=14,879; 899,071 total app uses) were analyzed to assess the validity of commonly given habit formation advice to meditate at the same time every day, preferably in the morning.

**Methods:**

First, the change in probability of meditating in 4 nonoverlapping time windows (morning, midday, evening, and late night) on a given day over the first 180 days after creating a meditation app account was calculated via generalized additive mixed models. Second, users’ time of day preferences were calculated as the percentage of all meditation sessions that occurred within each of the 4 time windows. Additionally, the temporal consistency of daily meditation behavior was calculated as the entropy of the timing of app usage sessions. Linear regression was used to examine the effect of time of day preference and temporal consistency on two outcomes: (1) short-term engagement, defined as the number of meditation sessions completed within the sixth and seventh month of a user’s account, and (2) long-term use, defined as the days until a user’s last observed meditation session.

**Results:**

Large reductions in the probability of meditation at any time of day were seen over the first 180 days after creating an account, but this effect was smallest for morning meditation sessions (63.4% reduction vs reductions ranging from 67.8% to 74.5% for other times). A greater proportion of meditation in the morning was also significantly associated with better short-term engagement (regression coefficient *B*=2.76, *P*<.001) and long-term use (*B*=50.6, *P*<.001). The opposite was true for late-night meditation sessions (short-term: *B*=–2.06, *P*<.001; long-term: *B*=–51.7, *P*=.001). Significant relationships were not found for midday sessions (any outcome) or for evening sessions when examining long-term use. Additionally, temporal consistency in the performance of morning meditation sessions was associated with better short-term engagement (*B*=–1.64, *P*<.001) but worse long-term use (*B*=55.8, *P*<.001). Similar-sized temporal consistency effects were found for all other time windows.

**Conclusions:**

Meditating in the morning was associated with higher rates of maintaining a meditation practice with the app. This is consistent with findings from other studies that have hypothesized that the strength of existing morning routines and circadian rhythms may make the morning an ideal time to build new habits. In the long term, less temporal consistency in meditation sessions was associated with more persistent app use, suggesting there are benefits from maintaining flexibility in behavior performance. These findings improve our understanding of how to promote enduring healthy lifestyles and can inform the design of mHealth strategies for maintaining behavior changes.

## Introduction

Unhealthy lifestyle factors account for 53% of all years of life lost prior to the age of 65 years [1]. Effective behavior-altering interventions can reduce these preventable deaths, but most interventions do not reach their potential and produce only limited, short-lived effects, as individuals often struggle to maintain new health behaviors over time [1-3]. A widely recognized approach for maintaining healthy behaviors is to establish a habit, which is defined as a frequently repeated behavior occurring as a reflexive response to a contextual cue [4]. Interventions focused on forming healthy habits have been pursued in many fields but have struggled to establish enduring behavioral change [5], indicating that the factors impacting long-term behavior change are poorly understood.

Mobile health (mHealth), defined by the World Health Organization as the use of mobile and wireless technologies to support the achievement of health objectives [6], is an area where establishing habits can be particularly challenging. While mHealth encompasses the entire intersection of information and communication technologies [7], it often takes the form of smartphone apps [8], which have well-documented struggles with maintaining user engagement [9-13]. Design features may contribute to this phenomenon, since apps routinely use notifications to increase engagement [14], which may unintentionally create a technological dependency that counters habit formation [15].

Meditation apps are an mHealth tool with the potential to significantly improve population health outcomes. Research consistently shows that meditation practitioners better manage stress than nonpractitioners [16-19], with positive impacts noted across a wide range of populations, including school children [20], undergraduate college students [21], parents [22], veterans with posttraumatic stress disorder [23], women undergoing cancer treatment [24], and workers experiencing burnout [25]. Meditation with an app has also been associated with improvements in physical health and mental health conditions [26] such as depression and anxiety [27]. Consequently, meditation has become increasingly popular, as indicated by a study showing a 14.2% increase in the practice of meditation from 2012 to 2017, making it the fastest growing behavioral health trend accounted for by the US Centers for Disease Control and Prevention [28]. Additionally, a 2022 analysis projected a 41.01% compound annual growth rate in the meditation app market from 2020 to 2027 [29], although recent reductions in demand since a peak during the COVID-19 pandemic may interrupt this trajectory [30].

While the increasing popularity of meditation and meditation apps suggests great potential, only a handful of studies have investigated factors that impact whether meditation app use is maintained over time. Stecher et al [31] found that participants who adhered to an anchoring strategy (also known as piggybacking), where app-assisted meditation was linked with existing daily habits, had a more persistent meditation practice. Lea et al [32] highlighted the role of regular routine in building meditation habits. In addition to these empirical findings, a common suggestion given by practitioners and teachers for maintaining a meditation practice is to meditate at a specific time that is convenient for the practitioner’s life and can be replicated daily [33]. Meditating immediately upon waking in the morning is often suggested as an ideal time for this activity, in order to avoid distractions or obstructions [34]. Prioritizing morning meditation has been observed in a previous trial, where participants who most successfully adhered to an anchoring strategy for meditation almost exclusively used morning anchors [31]. Furthermore, research in other domains shows that performing a target behavior in the morning (eg, taking diabetes medication [35] or engaging in a stretching routine [36]) leads to stronger habits. Similarly, specific action plans for fruit and vegetable intake were most effective when they focused on the morning [37]. 

The recommendation to meditate at the same time every day is more questionable. Theoretically, habits are characterized by engagement under similar conditions, such as time, place, and antecedent stimuli [38,39]. However, recent studies have found that rigid routines can be counterproductive for maintaining physical exercise, and that in some scenarios, flexibility in behavior timing is more desirable [40,41].

Investigating time of day (TOD) preferences for meditation and other health behaviors has been limited by the availability of detailed behavioral data. To date, habits are typically measured using cross-sectional surveys containing instruments such as the Self-Report Habit Index and its derivatives [42,43], which include items similar to “Behavior X is something I do automatically” and “Behavior X is something that belongs to my (daily, weekly, monthly) routine.” Such measures do not consider daily assessments of behavior timing and have questionable accuracy [44] since they require responders to consciously describe what is theorized as a nonconscious process. Alternatively, mHealth apps, despite the shortcomings mentioned above, have features that are opportune for investigating behavioral TOD preferences, including their ability to collect objective behavioral data at an unprecedented scale [45]. In this way, mHealth apps offer a revolutionary way to examine habits in daily life [46].

In this study, we test the utility of the commonly offered advice to maintain behavior change by meditating at the same time every day, preferably in the morning. We do so by examining how the maintenance of mHealth meditation app use is impacted by TOD preferences and the temporal consistency of meditation sessions. Since most contextually cued habits are performed at approximately the same time and in the same location each day [47,48], measures of temporal consistency can be assumed to be associated, in part, with habitual behavior [35,49]. Therefore, we use novel analytic methods based on intensive, objective behavioral measures to reveal temporal characteristics that may be associated with habit formation.

## Methods

### Sample Description

Data for this analysis were provided by Calm, a popular meditation app that had >2 million paying subscribers at the time of data collection. Calm provided a sample of 14,958 randomly selected users who created their account in 2017 and used the app at least 8 times over the first 2 months after their account was created. This last criterion eliminated participants who did not establish an average of at least one use per week in the time immediately after their account was created. The sample was selected such that the timing during which participants last used the app was spread evenly over the 2 years after account creation. Sample demographics were not provided other than estimates of age and gender made via a Calm proprietary prediction algorithm, which were not used in our analyses.

### Ethics Approval

The Chapman University Institutional Review Board provided an exemption from review (IRB-21-88) for this project.

### Measures

#### Meditation Sessions

The data consisted of 6,096,560 total sessions using the app, each classified as 1 of 8 types based on features of the app: Body, Breathe, Masterclass, Meditation, Mood, Music, Sleep, Soundscape, and Spark. For this analysis, we used only those sessions that were (1) classified as using the Meditation feature, (2) were tagged as completed, (3) were ≥3 minutes and ≤60 minutes in duration, and (4) occurred within the first 180 days after creating an account. This left a total of 899,071 unique meditation sessions for analysis.

#### Enrollment Day

For each participant, the account creation day was considered day (*d*)=1, and all subsequent meditation sessions were characterized by the number of days since this date. For example, if a participant created their account on March 1, 2017, and used the app for the first time on March 3, 2017, this session would be associated with *d*=3.

#### TOD Preference

For each participant, TOD preference was calculated as the proportion of total meditation sessions that began in each of 4 nonoverlapping time windows: morning (4 AM-9:59 AM), midday (10 AM-3:59 PM), evening (4 PM-9:59 PM), and late night (10 PM-3:59 AM).

#### Temporal Consistency

To quantify regularity in the timing of meditation sessions, the information entropy of meditation sessions was calculated, which captures the “surprise” or “uncertainty” in meditation timing. The entropy formula was as follows:




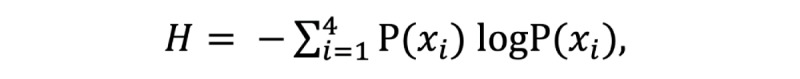




where *H* is the entropy and P(x_i_) is the empirically calculated probability of meditating during time window *i*, where *i* = {morning, midday, evening, late night}. If P(x_i_) = 0, then the summand 0 ∙ log(0) is set to 0, which is true in the limit. *H* can take values between 0 and log 0.25 (=1.38), with 0 representing meditating exclusively in 1 time window (ie, temporal consistency) and log 0.25 representing equal probability of meditating during each of the 4 windows (ie, no TOD preference).

Following previous work demonstrating that the effects of consistency in the timing of behavior can differ based on the time horizon at which outcomes are measured [40], statistical analyses were performed for both short- and long-term outcomes.

#### Short-Term Maintenance (Outcome 1)

Short-term maintenance of meditation behavior was quantified as the number of meditation sessions completed within the sixth and seventh month of an account’s existence and is denoted from here on as M67. The duration of this measure was selected to match the 8 uses in 2 months screening criterion for the sample.

#### Long-Term Maintenance (Outcome 2)

Long-term maintenance of meditation behavior was quantified as the last day prior to the data being pulled on which a meditation session was observed and is denoted as LS.

#### Account Creation Date

Data for this analysis was pulled on a single date in July 2021, so when considering the LS outcome, participants who created an account early in the 2017 calendar year will have a greater opportunity for larger LS values than those whose account was created later in the year. Therefore, regression analyses that consider the LS outcome include the date in 2017 on which the account was created as a covariate. This metric, called the account creation date (ACD), was an integer ranging from 1 for accounts created on January 1, 2017, to 365 for accounts created on December 31, 2017.

### Statistical Analysis

#### Overview

In addition to reporting summary statistics, we performed 3 statistical analyses, which are fully detailed below. Our first analysis, probability of meditation over time, was designed to provide a bird’s-eye view of meditation frequency at various times of day during participants’ first 180 days with an account. The second analysis, effects of TOD preference and temporal consistency, is a regression-based approach that investigates our primary research objective of determining whether 2 components of common meditation advice (ie, meditating in the morning and at the same time every day) are associated with a consistent meditation practice. The last analysis, TOD preferences over time, more comprehensively characterizes participants by using a clustering approach to identify common typologies concerning their TOD preferences over time. All analyses were performed for both short-term (M67) and long-term (LS) outcomes. We did not include age or gender in the statistical models, since they were estimated by a proprietary algorithm that we were not able to validate.

#### Summary Statistics

The mean (SD) was calculated for each measure.

#### Probability of Meditation Over Time

The probability of a meditation session occurring on any given day over the first 180 days after participants created their account was calculated via a generalized additive mixed model (GAMM) with a logit link function. Meditation probability was the dependent variable and, to control for repeated measures, participant ID was specified as a random-intercept effect. The independent variable was *d* (ie, enrollment day), which was modeled with an 8-knot, cyclical cubic spline smoothing function, a nonparametric approach that allows for a nonconstrained fit of daily probabilities. This process was repeated for each of the morning, midday, evening, and late-night TOD windows. To investigate differences associated with the frequency of meditation, all analyses were then performed separately for participants in the first (bottom) and fourth (top) quartiles of both the M67 and LS outcomes. For each GAMM, the difference in the predicted probability on *d*=1 and *d*=180 was calculated in both raw units and as a percent change.

#### Effects of TOD Preference and Temporal Consistency

Linear regression models were fit to determine the effects of TOD preference and temporal consistency on short- and long-term outcomes. Each outcome was set as the dependent variable, and temporal consistency and the total number of meditation sessions over the first 60 days after account creation were independent variables. TOD preference was also included as an independent variable, but because the 4 TOD measures are linearly dependent and therefore highly correlated, a separate regression model was built for each TOD window. For the LS outcome, the ACD was also included in the model.

#### TOD Preferences Over Time

To examine how TOD preference evolved over time, each participant’s data was stratified into 25 weeks (from *d*=1 through *d*=175). Since TOD preference is defined by a probability for each TOD window, this process produced four 25-week trajectories for each participant, 1 per TOD window. To identify archetypal trajectories among participants, a distinct *k*-means clustering was performed for each of the TOD trajectories. As will be shown in the Results section, an analysis of the total sum of square error resulted in 4 clusters, labeled as clusters 1 to 4, being selected for this analysis. Clusters 1 to 4 were qualitatively similar across each of the 4 TOD windows, which allowed each participant to be characterized as belonging to 1 of 256 possible TOD trajectory profiles (4 cluster options for each of the 4 TOD windows; 4^4^=256 total options). The 10 TOD trajectory profiles associated with the highest average LS and M67 outcomes were identified, and the proportion of cluster assignments within these trajectories was calculated.

## Results

### Summary Statistics

Table 1 illustrates summary statistics for the variables used in subsequent analyses, with all measures averaged over each of the 14,958 participants. The highest proportion of meditation sessions occurred during the morning and evening TOD windows, and entropy was relatively high, meaning that participants tended to vary the time window during which their meditation occurred. Participants averaged 11 meditation sessions during the sixth and seventh months of their enrollment and had their last meditation session 656 days after their account was created.

**Table 1:** Summary statistics for measures used in the study.

Statistics			Values, mean (SD)
**Time of day proportion**			
	Morning	0.31 (0.29)	
	Midday	0.17 (0.19)	
	Evening	0.29 (0.25)	
	Late night	0.22 (0.25)	
Entropy			0.86 (0.35)
M67^a^ value			11.04 (18.36)
LS^b^ value			656.40 (493.07)

^a^M67: months 6 and 7 outcome.

^b^LS: day of last session outcome.

### Probability of Meditation Over Time

**Figure 1:**
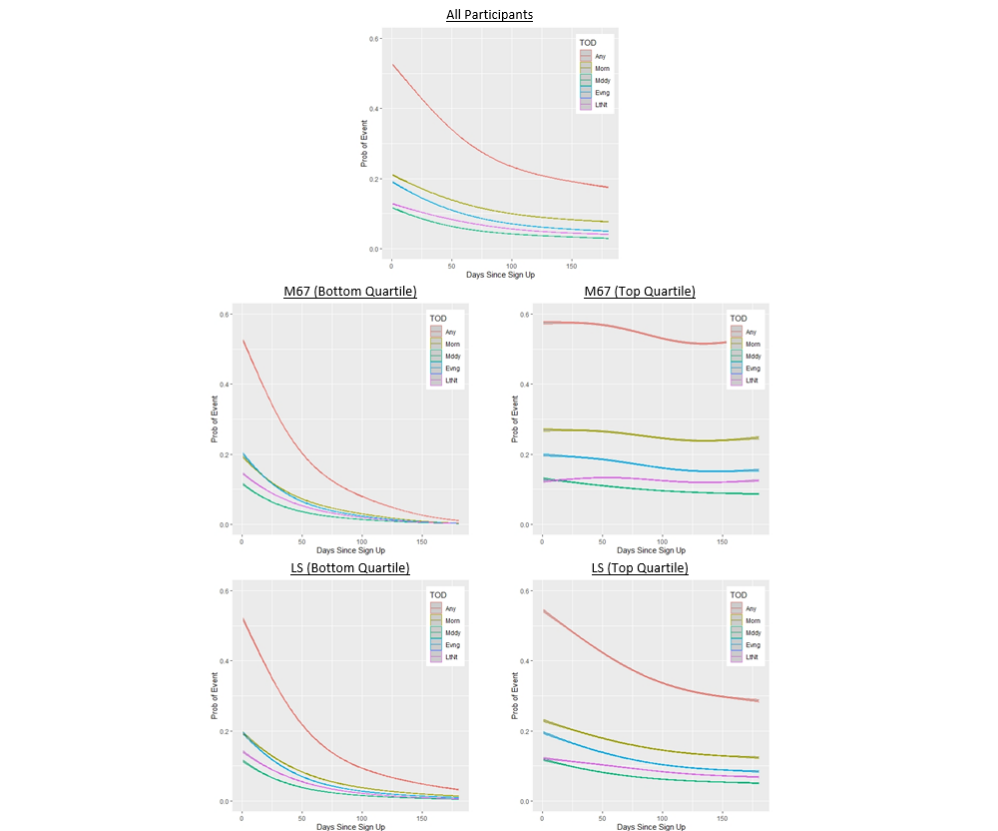
Predicted probabilities of meditation from generalized additive mixed models based on enrollment day. Models were fit separately for all sessions plus those occurring in the morning, midday, evening, and late night. This procedure was repeated for subsets of participants representing the bottom (first) and top (fourth) quartiles for both outcomes. Any: all sessions; Evng: evening; LS: day of last session; LtNt: late night; M67: months 6 and 7; Mddy: midday; Morn: morning; Prob: probability.

Figure 1 illustrates the results of the GAMMs that estimated the probability over time of meditating on a given day. For all participants, there was just over a 50% probability of meditating immediately after creating their account, with this value quickly decreasing over time. This pattern was consistent for participants in the bottom quartile of the M67 and LS outcomes and in the top quartile of the LS outcome, albeit with a slower decay in meditation probability for the latter group. In contrast, the probability of daily meditation for participants in the top quartile of the M67 outcome was relatively constant over time. Notably, for all participants, including those in the top and bottom quartiles of the outcome measures, the probability of meditating immediately after creating the account was relatively consistent (between 50% and 60%) for the earliest days of enrollment.

Figure 1 also illustrates differences in meditation probability according to TOD window. For participants in the top quartile of both outcome measures, the probability of meditating was highest in the morning, followed by the evening, late night, and midday. Similar results were seen for participants in the bottom quartile of the outcomes, but with minimal differences between morning and evening probabilities and a convergence of probabilities for all TOD windows to approximately zero when examining later enrollment. The latter effect is expected for participants with relatively poor outcomes. Table 2 quantifies these effects by showing the percent change in probability from *d*=1 to *d*=180 for all outcomes and participants. The percent change for participants in the top quartile on both measures was considerably smaller for morning and late-night meditation than for meditation that occurred during midday or evening. For example, morning and late-night reductions were 8.4% and 2.3%, respectively, for M67 compared with 33.5% and 21.8%, respectively, for midday and evening meditation. In other words, participants who meditated at a higher frequency were more persistent in their morning and late-night meditation than they were at other TODs. A similar result was found for the LS outcome, but differing probability reductions by TOD were not observed for participants in the bottom quartile of either outcome.

**Table 2:** Percent change in predicted probability of meditating on day 1 to day 180 for each time of day in each generalized additive mixed model.

	Any Time, %	Morning, %	Midday, %	Evening, %	Late Night, %
All participants	–66.5	–63.4	–74.5	–73.5	–67.8
M67^a^ bottom quartile	–97.8	–98	–98	–98.4	–98
M67 top quartile	–7.3	–8.4	–33.5	–21.8	–2.3
LS^b^ bottom quartile	–93.5	–92.6	–95.4	–94.7	–95.1
LS top quartile	–47.2	–46	–56.8	–56.7	–43.5

^a^M67: months 6 and 7 outcome.

^b^LS: day of last session outcome.

### Effects of TOD Preference and Temporal Consistency

Table 3 summarizes the results of multiple regression models used to assess the relationships between the number of meditation sessions, temporal consistency, and TOD preference on both outcomes. In the table, the morning, midday, evening, and late night columns each represent a single regression of the form 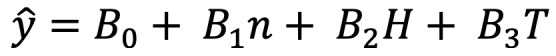, where 
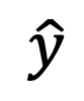
 is the M67 or LS outcome, *n* is the number of meditation sessions, *H* is the entropy, and *T* is 1 of the 4 TODs. As expected, there was a significant positive relationship between the number of meditation sessions over the first 180 days and both outcome measures, such that each additional meditation session was associated with approximately 0.4 more meditation sessions during months 6 and 7 and the last observed meditation session occurring approximately 4 days later. The effect of temporal consistency was reversed for the 2 outcomes, with more temporal consistency (ie, a lower value for *H*) producing better outcomes for M67 and worse outcomes for LS (both associations were statistically significant). A greater proportion of meditation in the morning was significantly associated with better short- and long-term outcomes. For example, a 10% increase in the proportion of meditation performed in the morning was associated with 0.28 more meditation sessions during months 6 and 7 and the last observed meditation session occurring approximately 5.5 days later. The opposite was true for late-night meditation, where a greater proportion of these sessions was associated with poorer meditation outcomes. The standardized regression coefficients indicate that for both outcomes, the associations with temporal consistency and TOD preferences had relatively similar magnitudes. However, the effect of the number of sessions was roughly 10 times as large for the M67 outcome and 4 to 5 times as large for the LS outcome, relative to temporal consistency and TOD preferences.

**Table 3:** Results of multiple regression used to predict each outcome. The outcomes for months 6 and 7 and day of last session are expressed in different units, so their regression coefficients are not comparable. *B* represents the fitted regression coefficient, and β represents the standardized regression coefficient.

Outcomes		Morning				Midday				Evening				Late Night		
		*B* (SE)	β	*P* value	*B* (SE)		β	*P* value	*B* (SE)		β	*P* value	*B* (SE)		β	*P* value
**Months 6 and 7 outcome**																
	Sessions (n)	0.41 (0.007)	.45	<.001	0.42 (0.007)		.46	<.001	0.42 (0.007)		.45	<.001	0.42 (0.007)		.45	<.001
	Temporal consistency (*H*)	–1.64 (0.40)	–.03	<.001	–2.32 (0.41)		–.04	<.001	–2.12 (0.39)		–.04	<.001	–2.06 (0.39)		–.04	<.001
	TOD^a^ proportion (*T*)^b^	2.76 (0.47)	.04	<.001	1.31 (0.73)		.01	.07	–1.44 (0.53)		–.02	.007	–2.77 (0.53)		–.04	<.001
**Day of last session outcome**																
	Sessions (n)	3.87 (0.21)	.16	<.001	3.97 (0.20)		.16	<.001	4.00 (0.20)		.16	<.001	3.94 (0.20)		.16	<.001
	Temporal consistency (*H*)	55.8 (11.8)	.04	<.001	51.2 (12.0)		.04	<.001	46.9 (11.6)		.03	<.001	48.1 (11.6)		.03	<.001
	TOD proportion (*T*)^b^	50.6 (13.9)	.03	<.001	–29.5 (21.4)		–.01	.17	2.98 (15.6)		0	.85	–51.7 (15.7)		–.03	.001
	Account creation date	–0.44 (0.04)	–.09	<.001	–0.44 (0.04)		–.10	<.001	–0.44 (0.04)		–.10	<.001	–0.44 (0.04)		–.09	<.001

^a^TOD: time of day.

^b^Time of day proportion (*T*) values were input at proportions ranging from 0 to 1. Therefore, their regression coefficients represent the difference in dependent variables between no meditation (*T*=0) and all meditation (*T*=1) during a given time of day window.

### TOD Preferences Over Time

**Figure 2:**
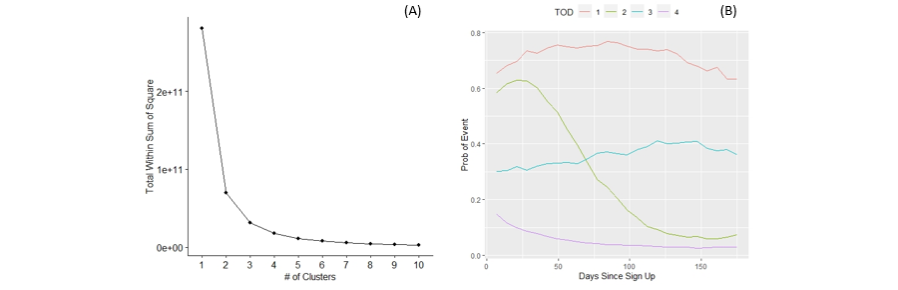
Results of clustering analysis for weekly summaries of the probability of meditating in the morning. (A) Total sum of square error as a function of the number of clusters (ie, *k*) input to a *k*-means clustering procedure, which indicates 4 four clusters should be used. (B) Prototypes (ie, cluster centroids) from each of the 4 clusters. These results are for the morning, but similar results were found for all other time of day windows. Prob: probability; TOD: time of day.

Based on the total sum of squared error (Figure 2 and Table 4), participants’ proportion of morning meditation was stratified into 4 clusters. Clusters 1 and 2 indicate a high proportion of morning meditation at the outset of enrollment, with this level being sustained over time in cluster 1 and decreasing with time in cluster 2. Cluster 3 represents a consistent, moderate rate of meditation over time, while cluster 4 is indicative of a low level of meditation throughout the observation period. The clusters identified in the other TOD windows were qualitatively similar to those shown in Figure 2. Table 4 shows the number of individuals assigned to each cluster for each TOD. Cluster 4 was by far the largest cluster, with other clusters containing roughly 5 to 10 times fewer participants.

Because clusters are consistent across TODs, a 4-component TOD trajectory profile can be built to summarize each participant’s TOD preferences over time. We denote each profile as *m-d-e-l*, where {*m,d,e,l*}∈{1..4} corresponds to one of the 4 cluster groups shown in Figure 2 for morning, midday, evening, and late night, respectively. For example, a 1-4-4-3 cluster profile corresponds to a participant who had a consistently high proportion of morning meditation, low levels of midday and evening meditation, and a consistently moderate proportion of late-night meditation. Table 5 illustrates the 10 TOD trajectory profiles (minimum 50 participants per profile) with the highest average value for both outcomes, which represents 1163 participants for the M67 metric and 1012 participants for the LS metric. For each TOD window, we also calculated the proportion of each cluster included in these top-performing profiles (Table 6). Notably, cluster 2 was severely underrepresented, indicating that participants who began with a high proportion of meditation within any TOD window and then dramatically dropped off did not fare well for either outcome measure. Additionally, for every outcome/TOD combination, a considerably higher proportion of participants had TOD trajectory profiles characterized by cluster 3 rather than cluster 1, indicating that sustaining a moderate frequency of meditation at a given TOD led to better outcomes than sustaining a high frequency.

**Table 4:** Summary of the number of participants associated with each cluster type for each time of day.

Time of day	Cluster 1, n	Cluster 2, n	Cluster 3, n	Cluster 4, n
Morning	1532	2171	1847	9329
Midday	751	1911	2088	10129
Evening	1006	2398	2376	9099
Late night	899	1988	1827	10165

**Table 5:** The 10 cluster profiles (morning–midday–evening–late night) with the highest average values for the months 6 and 7 and day of last session outcomes, with a minimum of 50 participants in each profile.

Highest values for number of sessions in months 6 and 7				Highest values for the day of last session		
Cluster profile	Participants, n	Number of sessions	Cluster profile		Participants, n	Day of last session
3-3-3-3^a^	52	41.3	1-4-4-3^a^		53	962.2
1-4-4-3^a^	53	38.2	3-3-3-3^a^		52	958.6
3-4-4-1	68	33.1	3-4-3-3^a^		81	933.3
3-4-3-3^a^	81	33.1	2-3-3-4		69	909.9
1-3-4-4	250	30.2	4-4-3-1		190	902.6
3-3-4-3	58	29.6	3-4-3-4		113	900.3
3-3-3-4	191	29.1	4-1-3-4		159	891.1
1-4-3-4^a^	87	28.3	4-3-3-3^a^		103	891.0
4-4-1-3	220	27.3	1-4-3-4^a^		87	881.3
4-3-3-3^a^	103	27.3	4-3-1-4		105	875.4

^a^These cluster profiles are present in both lists.

**Table 6:** Weighted proportion of each time of day window in the top-ten cluster profiles outlined in Table 5. As an example, the first row under “Day of last session” shows results associated with the morning time of day window and the day of last session outcome. The results indicate that for the 1012 participants summarized in the right three columns of Table 5, 140 (53 {1-4-4-3} profiles + 87 {1-4-3-4} profiles), or 14%, were assigned to cluster 1 for the morning window, 7% were assigned to cluster 2 for the morning window, 24% were assigned to cluster 3, and 55% to cluster 4. The remaining rows summarize results for other time of day windows and outcomes.

			Cluster 1, n (%)		Cluster 2, n (%)		Cluster 3, n (%)		Cluster 4, n (%)
**Months 6 and 7 (n=1163)**									
	Morning	390 (34)		0 (0)		450 (39)		323 (28)	
	Midday	0 (0)		0 (0)		654 (56)		509 (44)	
	Evening	220 (19)		0 (0)		514 (44)		429 (37)	
	Late night	68 (6)		0 (0)		567 (49)		528 (45)	
**Day of last session (n=1012)**									
	Morning	140 (14)		69 (7)		246 (24)		557 (55)	
	Midday	159 (16)		0 (0)		329 (33)		524 (52)	
	Evening	105 (10)		0 (0)		854 (84)		53 (5)	
	Late night	190 (19)		0 (0)		289 (29)		533 (53)	

## Discussion

### Principal Results

In this paper, we examined the effects of TOD preferences and temporal consistency on both short-term and long-term indicators of sustaining an mHealth meditation practice. This analysis was undertaken to assess the appropriateness of common advice for establishing a meditation habit, namely, to meditate at the same time every day, preferably in the morning.

Each of the 3 statistical analyses presented herein supports the conclusion that meditating in the morning is beneficial for sustaining engagement with the mHealth app. First, we observed that the decrease in the probability of meditating over the first 180 days after creating an account was statistically smaller for morning meditation sessions (63.4% reduction) than for other times of day (reductions ranged from 67.8% to 74.5%). This was especially true for participants whose outcome measures were in the top quartile (ie, > the 75th percentile) of all measures. Second, when regressing both short-term and long-term outcome variables on TOD proportion, a 10% increase in the proportion of all meditation sessions performed in the morning was associated with 0.28 more meditation sessions during months 6 and 7 and the last observed meditation session occurring approximately 5.5 days later. Similar-magnitude decreases in these 2 outcomes were associated with increases in the proportion of meditation sessions performed in the late night, and no effect was found for the proportion of meditation sessions performed in midday or evening. Lastly, the positive impact of morning meditation was also seen in the cluster analysis, where the top 10 most-observed cluster profiles contained fewer instances of consistent low levels of meditation (cluster 4) and greater instances of sustained meditation (clusters 1 and 2) for the morning TOD window than for the other windows, particularly for the M67 outcome. This consistent finding across multiple analyses supports the commonly provided advice of establishing a consistent meditation practice in the morning and suggests that future meditation interventions should target morning meditation for helping users attain the corresponding health benefits.

Further, our findings show that a high intensity of meditation practice at the start of using the app was generally not sustainable. This can be seen by the lack of cluster 1 representation in the TOD trajectory profiles in Table 6, which indicates that people with the best short- and long-term outcomes were not able to sustain an exceptionally high level of meditation. Instead, they either sustained a more moderate probability of meditating in a given TOD window (cluster 3) or began with a high probability but then decayed to a probability near zero (cluster 2). Among users that started with high intensity (cluster 1), doing so in the morning was more sustainable than any other TOD. In other words, initial high intensity did not prove to be a good method for sustained app meditation practice, but if one were to meditate zealously, it would be better to do so in the mornings.

These findings should be expanded upon in future work by using ecological momentary assessments or environmental sensors to measure contextual cues that will help to better determine if and when meditation has become habitual.

### Limitations

There were several limitations to this study. First, generalizability may be an issue given our limited access to demographic and other contextual information, and our findings may not extend to other behaviors, since app-based meditators generally tend to be female with low racial diversity [28,50]. Second, our findings do not account for any meditation sessions not performed with the app, which could bias our results if more experienced meditators eventually find the app to be extraneous and unnecessary. Third, we do not account for other app features (eg, Sleep Stories) that may impact participants’ app usage. Lastly, we use temporal consistency as a proxy for routine app usage, but not all routines and habits will be performed at approximately the same time each day, as they may be contingent upon a time-varying cue (eg, meditating after a work shift that varies from day to day).

### Comparison With Prior Work

One possible explanation for the success of morning meditations could be the influence of the circadian rhythm of cortisol. In attempting to understand the development of healthy habits at various points throughout a circadian cycle, Fournier et al [36] found that morning practitioners were the quickest to habituate a targeted behavior. Further, cortisol levels played a significant role in determining the time for the healthy behavior to become a habit, with cortisol levels being higher in the morning than in the evening. According to Adan et al [51], preferences toward activity at certain times in the day indicate a circadian typology, and those with a preference for morning activity (ie, a morning typology) tend to exhibit healthier overall habits. This is concurrent with findings from Taylor et al [52], who observed better academic performance in college students with morning typology preferences. A suggested (but not yet tested) explanation for this phenomenon is that contexts and behaviors in the morning are more stable and, therefore, more conducive to habit development [35]. Beyond the human experience, even snails have been shown to learn more efficiently in the morning [53]. While our findings might support the conclusion that habit formation is best for those with a morning typology, TOD preferences should not be the only indicator, as temporal consistency should also be taken into account.

Contrary to popular advice to meditate at the same time every day, this study indicates that temporal consistency in meditation timing was inversely related to a long-term meditation practice, although it was beneficial for short-term outcomes. This finding is consistent with recent work indicating that flexibility, rather than routine, led to better exercise outcomes. Beshears et al [40] found that incentivizing gym attendance only during a participant-selected 2-hour window led to less gym attendance than when participants were incentivized to attend at any time. The researchers hypothesized that this finding could be due to a bias in their sample, which was composed of technology managers who likely had less routine built into their day. However, it is notable that only 22% of participants selected a 2-hour window in the morning. Therefore, the poorer outcomes for routine incentives may reflect the suboptimal performance of nonmorning TOD preferences, as discussed above. Additionally, Berardi et al [41] found that stochastic exercise performance over the first 100 days of an intervention was associated with increased exercise throughout the remainder of the 1-year trial. Meta-flexibility was hypothesized as a potential mechanism for this finding, whereby participants who had the resilience to adjust their behavior in response to changing life circumstances were ultimately more successful at sustaining their exercise behavior.

### Conclusions

In conclusion, we have used novel analytic techniques to demonstrate that persistence in the use of an mHealth meditation app is most successful when meditation primarily occurs in the morning and when participants are not overly rigid to the point that no meditation occurs at other times of the day. These findings can be used by practitioners, researchers, and policy makers to develop behavioral interventions for meditation and other health-promoting behaviors that have the greatest chance of establishing healthy habits, thereby improving public health.

## Abbreviations

ACD: account creation date
GAMM: generalized additive mixed model
LS: day of last session
M67: months 6 and 7
mHealth: mobile health
TOD: time of day

## Data Availability

The data sets analyzed within the current study are not publicly available because they are proprietary.
